# Study on Failure Energy per Unit Area of Concrete Specimens Based on Minimum Energy Dissipation Theory

**DOI:** 10.3390/ma17010201

**Published:** 2023-12-30

**Authors:** Xinyu Liang, Zengbiao Wu

**Affiliations:** School of Electric Power, Civil Engineering and Architecture, Shanxi University, Taiyuan 030006, China; 18234488371@163.com

**Keywords:** concrete material, minimum energy consumption theory, damage area, destructive energy

## Abstract

In order to study the strength change of concrete specimens under different loading conditions, based on the principle of minimum energy dissipation, the damage energy per unit area of concrete was studied. By using finite element numerical simulation software for concrete specimens with different failure modes of tension, pressure, bending and torsion, a double-broken line damage constitutive model is adopted. The failure forms of concrete specimens under different loading conditions, as well as the failure area and failure energy of each specimen during loading, are simulated and analyzed. The failure energy per unit area under different failure modes was quantitively calculated, the relationship between the failure area and failure energy consumption under different failure modes was analyzed. The results show that, under different failure modes, the failure area of concrete specimens is different, the energy consumed during failure is different, and the strength is different. However, no matter how the failure mode changes during the failure process, the failure energy W per unit area remains constant and fluctuates in the range of 2.0~6.0 mJ/cm^2^, which is related to the physical properties of concrete itself.

## 1. Introduction

Many rock deformation behaviors and failure conditions have been described from the perspective of energy. You M Q et al. [1] analyzed the relationship between rock failure and energy, and revealed that the energy actually absorbed during the yield process is mainly dissipated by the friction work of shear slip inside the rock. Song X F et al. [2] studied the evolution law of energy during the rock failure process. In addition to the above, in different research studies, the strength of rocks has been examined from the perspective of energy [3,4,5,6]. However, due to the complex and heterogeneous nature of concrete materials, there are few studies on the deformation and failure of concrete materials based on the energy method. In the process of studying the strength of concrete materials, the strength factor is used as a parameter to study and analyze the stress field and displacement field of macroscopic cracks. Yang X H et al. [7] analyzed the stress field and displacement field of the crack tip of steel fiber concrete. The stress intensity factor of steel fiber bonded concrete crack tip was obtained. In addition, concrete strength has been analyzed and calculated by combining many methods [8,9,10,11,12,13] but, from the above perspective, it is often impossible to accurately solve, and there are errors. The advantage of studying strength from the perspective of energy is that energy is a physical quantity that runs through different structural levels, and it is more effective to study crack expansion or even fracture of multiphase composite materials such as concrete.

Damage theory has been widely used in the study of concrete [14,15,16,17]. Li Shuchun et al. [18] believe that, under the action of external factors, the cumulative deformation of materials will cause the development of internal damage of the structure, which will eventually lead to macroscopic cracks and even failure. A new segmental curve concrete compression damage evolution equation has been proposed, and the damage evolution equation of concrete with different strengths is also given. Yu Z W et al. [19] reviewed the research status of the concrete microscopic damage mechanism and random damage constitutive model, and proposed and verified the random damage constitutive model of concrete. Shen, J. et al. analyzed concrete damage through the theory of fracture energy regularization of high bench beams [20].

According to previous conclusions, the damage process is considered to be an irreversible energy conversion process. Based on the energy perspective, it is effective and reasonable to establish the relationship between damage variables and dissipated energy as a method to provide simulation analysis of concrete compression damage. In this study, the strength failure, failure form and failure process of concrete materials are studied by finite element numerical calculation from the angle of energy. The process of crack generation is simulated numerically to form the failure form of the failure surface, and the calculation method of the failure area of concrete under different stress states is proposed. In view of the dispersion of concrete strength and the difference of strength values under different load conditions, the factors affecting its strength are analyzed and studied, the mechanism of concrete strength change is explained, dissipative energy and failure surface are studied using the macroscopic mechanical properties of fracture mechanics [21], and the theory of concrete strength is explored. The reasons for the different intensities under different load conditions are explained from the point of view of failure area and energy.

## 2. Methods

Based on the principle of minimum energy dissipation [22], the finite element element of a unit volume of concrete specimen is taken as the consideration object, which produces deformation under the action of external force. Assuming that there is no heat exchange between the physical process and the outside world, the physical process is a closed system, and the total input energy generated by the external force is *U*, according to the first law of thermodynamics. This can be obtained as follows:

(1) $$U=U^{d}+U^{e}.$$

The energy of each part of the concrete specimen unit in the principal stress space can be expressed as:

(2) $$U^{d}=\int_{0}^{\varepsilon_{1}}\sigma_{1}d\varepsilon_{1}+\int_{0}^{\varepsilon_{2}}\sigma_{2}d\varepsilon_{2}+\int_{0}^{\varepsilon_{3}}\sigma_{3}d\varepsilon_{3}.$$

(3) $$U^{e}=\frac{1}{2}\sigma_{1}\varepsilon_{1}^{e}+\frac{1}{2}\sigma_{2}\varepsilon_{2}^{e}+\frac{1}{2}\sigma_{2}\varepsilon_{3}^{e}.$$

(4) $$\varepsilon_{i}^{e}=\frac{1}{E_{i}}[\sigma_{i}-\lambda (\sigma_{j}+\sigma_{k})].$$

*U* is the total work done by the main stress, the unit of dissipated energy *U^d^* is used to form the internal damage and non-recoverable deformation of the unit, and *U^e^* is the elastic strain energy that can be released by the unit.

**Figure 1:**
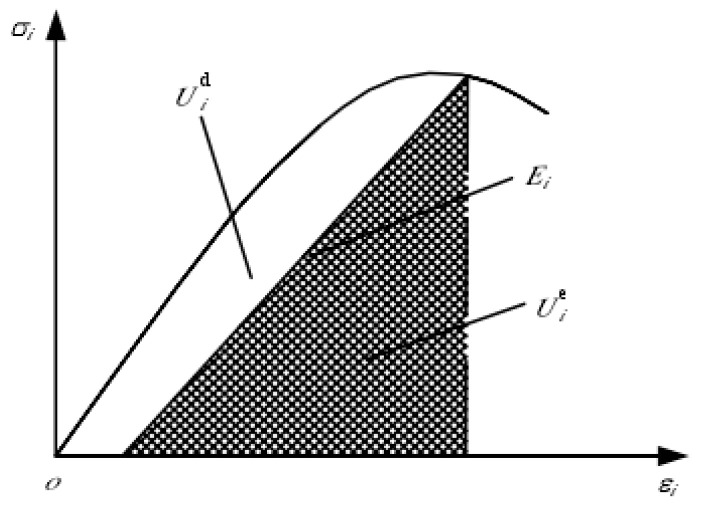
Quantity–value relationship between releasable strain energy and energy dissipation per unit volume.

Figure 1 shows the stress–strain curve of the concrete specimen and the quantifiable relationship between the releasable strain energy and energy dissipation per unit volume. The area *U^d^_i_* is the energy consumed when the unit is damaged, and the shadow area *U^e^_i_* is the releasable strain energy stored in the unit, which is the elastic strain energy released by the unit after unloading. *E_i_* is the unloading elastic modulus.

From the thermodynamic point of view, energy dissipation is irreversible, while energy release is two-way and reversible as long as certain conditions are met. The energy damage of the unit is defined as:

(5) $$\eta =\frac{U^{d}}{U^{c}}.$$

*U^c^* is the critical value of energy dissipation when the element strength fails, and is the material constant. In a certain stress state, *η* = 1 indicates loss of strength of concrete material.

The element “strength failure” is different from “failure”. The element is gradually damaged with the increase of strain until the dissipation energy of the element is close to the critical energy dissipation value of the element, but the concrete specimen does not necessarily suffer from failure. When the strain energy of a certain element can reach the energy required for the failure of the element, the element is damaged. The elastic strain energy stored in the elements is released in the form of elastic surface energy, and the overall failure of the specimen occurs when the cumulative failure of the elements reaches a certain number.

The sum of strain energy dissipation of element damage and strain energy dissipation of element failure is defined as dissipation energy. In this study, it is assumed that the concrete material is brittle and the failure is elastic fracture; that is, the energy provided by the outside world does not consider the energy dissipation caused by irrecoverable displacement, and dissipation energy is considered as failure energy.

## 3. Materials and Experiment

### 3.1. Materials

The specimen used in this study is a C15 concrete cylindrical specimen with a water–cement ratio of 0.40. The aggregate is Jing River and pebble with a particle size of 5~20 mm and the sand is middle sand from Xi’an Zhuohe River. The test was conducted after 28 days of maintenance under standard conditions. The specific dimensions are shown in Table 1.

### 3.2. Experimental Result

**Figure 2:**
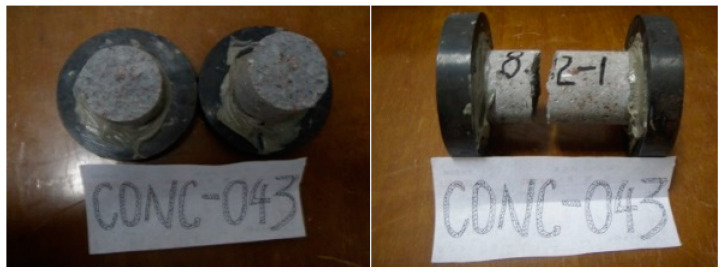
Failure under tensile stress.

The failure form of specimen Pull-1 under tensile force is shown in Figure 2. Under the action of pure tensile stress, the specimen is damaged along the cross section, and the failure fracture is relatively flat. No matter how large or small the ratio of length to diameter, the failure surface is uniformly parallel to the cross section.

The bending moment causes the bending failure of the specimen, the bending surface cracks along the cross section of the specimen, and the bending fracture is smooth and smooth.

**Figure 3:**
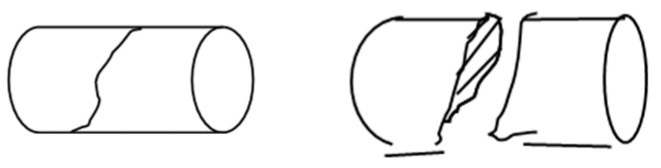
Twist-1 destruction diagram.

The failure of specimen Twist-1 is due to shear tensile failure, and the failure fracture is rough and uneven. As shown in Figure 3, its failure is from the outermost layer along the axis of the rod in about a 45 degree direction, and the failure surface is a spatial spiral twisted surface.

The concrete specimens subjected to pressure underwent various forms of failure, which are divided into long pressure, medium pressure and short pressure failure, in which the fixed length diameter ratio *λ* ≥ 10 is long pressure failure, 1 < *λ* > 10 is medium pressure failure, and *λ* ≤ 1 is short pressure failure. The failure surface of the specimen is shown in Table 2.

## 4. Numerical Simulation

### 4.1. Constitutive Model

**Figure 4:**
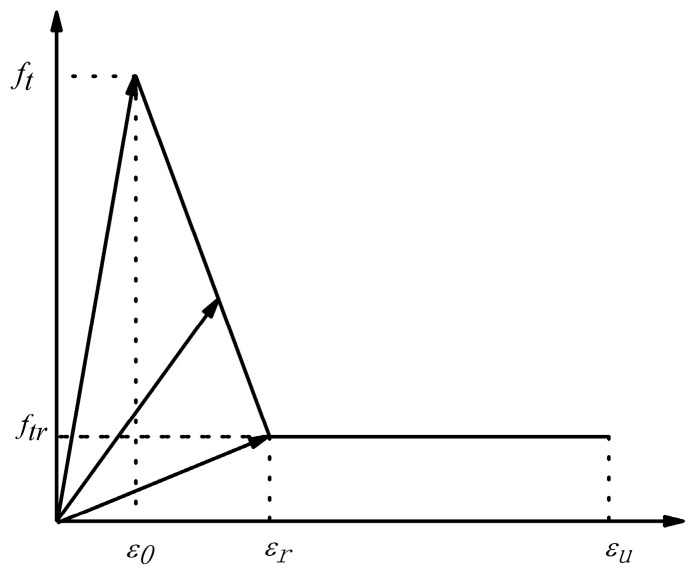
Bifold damage evolution model.

A double broken line damage evolution model was used in the numerical calculation. The influence of Poisson’s ratio on material damage was not considered in the calculation; only the elastic modulus damage was considered. The damage variable *D* of the elastic modulus is introduced, and the elastic modulus after damage is *E* = (*1* − *D*) *E*_0_. The change law of the damage variable with the strain is shown in the elastic bifold damage model, as illustrated in Figure 4.

(6) $$\tilde{E}=E_{0}\left(1-D\right)\;\left(0\leq D\leq 1\right).$$

(7) $$\sigma =\tilde{E}\varepsilon .$$

where *E*_0_ is the initial elastic modulus, $\tilde{E}$ is the residual elastic modulus, *σ* and *ε* are the nominal stress and nominal strain of the material, and the damage variable *D* is mainly determined by the double broken line damage evolution model. The expression of *D* is:

(8) $$D=\left\{\begin{array}{cc} 0 & \left(\varepsilon_{max}<\varepsilon_{0}\right)\\ 1-\frac{\eta -\lambda }{\eta -1}\frac{\varepsilon_{0}}{\varepsilon_{max}}+\frac{1-\lambda }{\eta -1} & \left(\varepsilon_{0}<\varepsilon_{max}\leq \varepsilon_{r}\right)\\ 1-\lambda \frac{\varepsilon_{0}}{\varepsilon_{max}} & {(\varepsilon }_{r}<\varepsilon_{max}\leq \varepsilon_{u})\\ 1 & \left(\varepsilon_{max}>\varepsilon_{u}\right) \end{array}\right..$$

(9) $$f_{tr}=\lambda f_{t}.$$

(10) $$\varepsilon =\eta \varepsilon_{0}.$$

(11) $$\varepsilon_{u}=\xi \varepsilon_{0}.$$

where, *f_t_* is the tensile strength, *f_tr_* is the residual tensile strength, *ε*_0_ is the principal tensile strain of *f_t_*, *ε_r_* is the residual strain when the unit tensile strength reaches the residual tensile strength. *ε_u_* is the ultimate tensile strain, and *ε_max_* is the maximum tensile strain corresponding to a load value at loading. *λ* is the residual tensile strength coefficient, *η* is the residual strain coefficient, and *ξ* is the ultimate tensile strain coefficient.

The bibroken line damage variable evolution model adopted in this paper considers that when the strain reaches its given limit value, the element begins to damage; that is, when the maximum tensile strain *ε_max_* < *ε*_0_ range of the mesoscopic element, the element is considered to have no damage. When the maximum tensile strain *ε_max_* > *ε_u_,* the mesounit is considered to be damaged. The first progressive damage occurs when the maximum tensile strain *ε_max_* is in the range of *ε*_0_ and *ε_r_*. The second progressive damage occurs when the maximum tensile strain *ε_max_* of the mesoelement is in the range of *ε_r_* and *ε_u_*.

The initiation, development and penetration of cracks are simulated, and the strength and failure process are analyzed from the perspective of energy. In the calculation, the load is evenly distributed on the upper surface of the specimen, which is divided into seven steps. The degree of elastic modulus reduction reflects the damage degree of the meso-concrete specimen under continuous loading, and the damage process of various phase materials of the meso-concrete can be described. In the calculation, the maximum tensile strain failure criterion is selected to carry out numerical simulation calculation of the concrete cylinder specimen.

In the loading process, when the maximum tensile strain of the element exceeds the ultimate tensile strain of the material, the element stiffness fails; that is, with the increase of the load, the elastic modulus of the element changes with the damage law of the bifold line. The static equilibrium equation of the element is nonlinear, and the equation is solved by the incremental method. As the damage degree of the elastic modulus varies with the increase of load, the solution process needs constant damage iteration, and the calculation amount is very large.

### 4.2. Numerical Model

The concrete is regarded as a random aggregate model of aggregate, mortar and interfacial three-phase materials. The establishment of the coordinate axis takes the center of the bottom surface of the cylinder specimen as the coordinate origin O, the axis of the cylinder specimen is the Z axis, the direction pointing to the top surface of the cylinder is the positive of the Z axis, and the plane perpendicular to the Z axis is the XY plane. The model adopts the Z direction constraint of the bottom surface and the midpoint constraint.

On the established 3D digital model of aggregate, the contact between aggregate and mortar is added by using the following process: In the software ANSYS, the concrete cylinder specimen is first established, and then the program is used to randomly generate aggregate spheres in the cylinder. Then the cylinder and aggregate spheres are separately assembled for BOOLEANS operation, so as to form a concrete specimen with hollow aggregate position. The program is used to read the position coordinates of the spheres to begin to generate aggregate spheres for the second time. A SOLID45 unit was selected for overall mesh division, and the material properties of the unit were judged by the aggregate projection mesh method. TARGEl70 and CONTAl74 unit types were selected to add contact units between the aggregate unit and the mortar unit.

**Figure 5:**
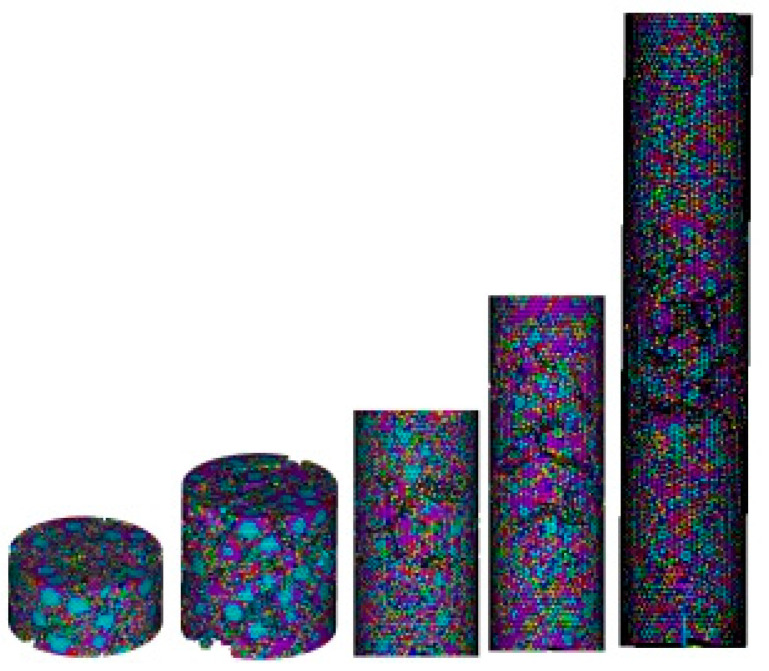
Failure diagram of the pressed specimens.

The numerical test simulated the tensile, compressive (in which the compression length-diameter ratio *H*/*R* = 1, 2, 4, 6, 10), bending and torsional damage, and the calculated model dimensions were consistent with those in Table 1. The concrete specimen models Pre-1, Pre-3 and Pre-5 are short pressure, medium pressure and long pressure, respectively, as shown in Figure 5.

When the short-pressure specimen is damaged under pressure, there are many cracks along the vertical direction, forming many longitudinal failure surfaces. After the specimen is damaged, it becomes fragmentary and the failure fracture is coarse. When the long-pressure specimen is under pressure, it displays instability failure. The failure surface develops and fails along the cross section, and the shape of the failure surface is very similar to that of the tension specimen Pull-1. When the medium-pressure specimen is under pressure, its behavior is between the transition stage of long-pressure and short-pressure, displaying shear failure. The failure surface gradually tilts along the cross section, forming a certain Angle *α* with the stress axis, and gradually tilts along the cross section. When the pure shear failure occurs (*α* = 45°), the failure surface is elliptical, and the level of the failure surface is between the long-pressure and the short-pressure. The data simulation results of tensile specimens, bending specimens and torsion specimens are consistent with the experimental results.

### 4.3. Numerical Simulation Calculation

It is necessary to go through the intermediate damage stage, from the initial stress to the final failure of the unit. From the initial loading to the final failure of the specimen, all of the unit damage strain energy dissipation and unit failure strain energy dissipation are added together to obtain the overall damage strain energy dissipation and failure strain energy dissipation of the specimen, and the sum of the overall damage strain energy dissipation and failure strain energy dissipation is the total energy dissipation of the specimen from loading to failure.

The method for determining the energy consumption of damaged elements is as follows: According to the average stress–strain of the unit, the strain energy of each unit at each load step can be calculated. After each load step is applied, according to the maximum tensile strain criterion, which units enter the damage stage and which units enter the failure stage can be judged. The difference between the strain energy of the unit before entering the damage stage and the strain energy of the unit after entering the damage stage is defined as the damage strain energy of the unit. The difference between the strain energy of the unit before the failure stage and the strain energy of the unit after the failure stage is defined as the failure strain energy of the unit.

**Figure 6:**
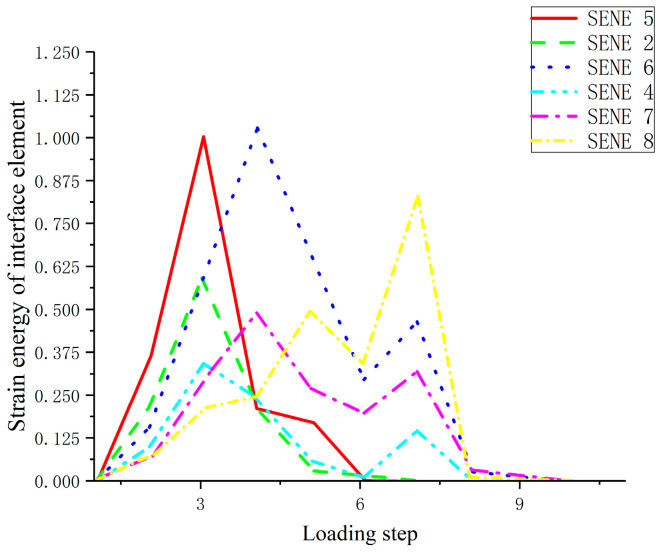
Strain energy variation diagram of interface element.

The strain energy of interface elements at different positions on the same section is used for analysis. With the increase of displacement, the strain energy first increases and then decreases. When different points reach the maximum strain energy, the displacement is different, and the change law of strain energy of elements at all surfaces is the same. This shows that the damage and consumption process of interfacial element strain energy with external force is complicated. With damage of the elastic modulus, its energy consumption increases continuously. However, due to the different positions of the interface elements, the values of the stored elastic strain energy of each element are different, but the change law of the strain energy of each element is the same. The fluctuation increases to the extreme value, and then all the strain energy stored in the unit is released, as shown in Figure 6.

**Figure 7:**
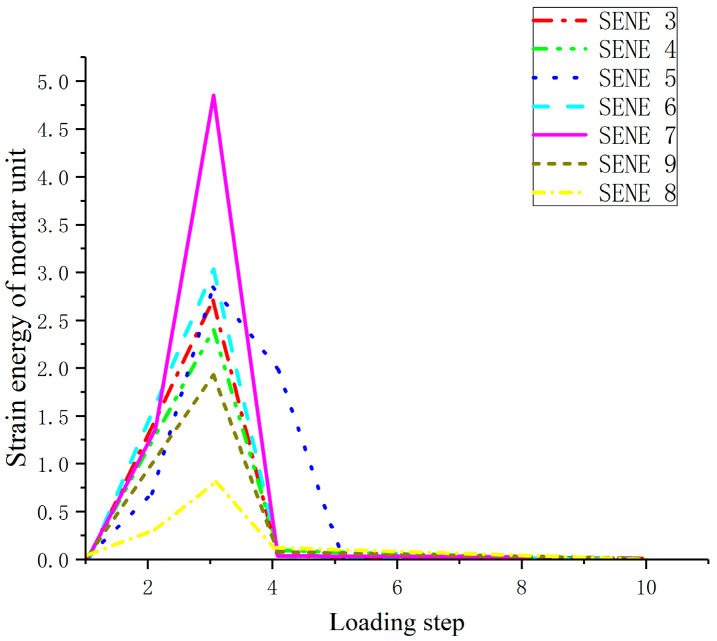
Strain energy variation diagram of mortar unit.

The strain energy of mortar units at different positions on the same section is also analyzed. With the increase of displacement, the strain energy first increases and then decreases, but when the maximum strain energy is reached at different points, the displacement is very close, indicating that the strain energy of mortar in the same section is synchronized with the damage and consumption of external forces, as shown in Figure 7.

**Figure 8:**
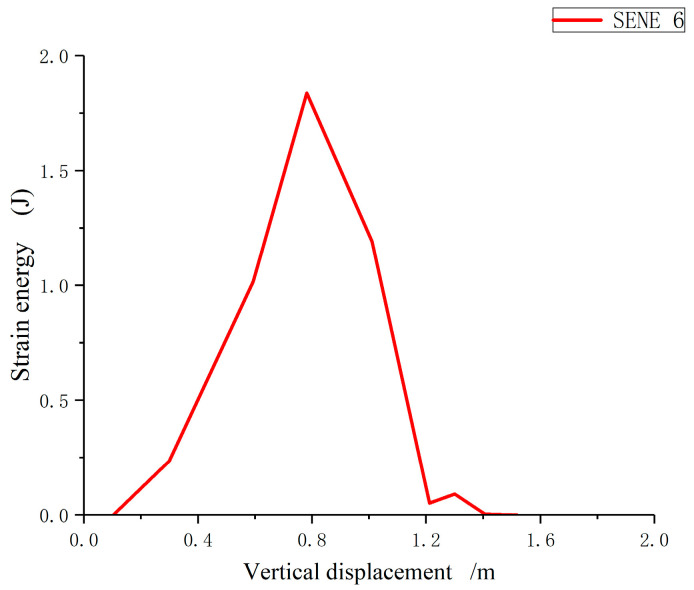
Strain energy variation diagram of the unit.

With the increasing load step, through the analysis of the changes in the stored strain energy of mortar and interfacial material units, the variation law of strain energy of specimens in the numerical calculation is basically the same, as shown in Figure 8: The strain energy of a unit increases with the increase of vertical displacement, and then the strain energy drops abruptly with the continuous increase of displacement, resulting in the failure of the unit.

The simulation of the numerical test calculated the tensile, compressive, bending and torsion damage, and calculated the number of interface and mortar kill elements, as well as the energy loss of interface and mortar. Since all model elements are divided into different sizes, the failure area of the specimen should be the number of mortar and interface kill elements multiplied by their respective surface area; the greater the failure area, the greater the failure energy.

## 5. Results and Discussion

### 5.1. Failure Area and Failure Energy

The relationship between failure area and energy is studied from the angle of crack energy release rate, and it is found that the larger the failure area is, the larger the energy consumption is. Tensile damage area is the smallest, the bending, long-pressure damage area is close to the tensile damage area, the medium-pressure damage area is greater than the long-pressure, and the torsional damage area is greater than the medium-pressure and less than the short-pressure damage area. The corresponding damage can be from small to large in the order of tension, bending, long-pressure, medium-pressure, torsion and short-pressure. It is concluded that the greater the strength of the specimen, the greater the area under stress, and the greater the energy of the external force to the surface energy of the specimen; that is, the larger the failure area is, the greater the failure energy of the specimen.

The failure forms of concrete specimens under different stress conditions are different, and the crack cracking type and development direction are different, resulting in different failure areas and different failure energies when the specimens are damaged, such as Pull-1, Pre-1, Pre-5 and Twist-1. However, under different loading conditions, the failure form is the same, the crack cracking type, development path and failure area are the same, and the failure energy of the specimens is the same, which determines the same strength, such as Pull-1, Pre-5 and Bending-1. According to the failure area obtained under different loading conditions, the failure energy is obtained, as shown in Table 3. The relationship between the new surface generated under different loading conditions and the external input energy of the linear elastic concrete specimen can be clearly seen in the table. At the same time, the energy lost in the rupture process of the concrete specimen minus the energy required for the deformation of the concrete specimen should be proportional to the new surface area. Generally, the surface area per unit volume is inversely proportional to the current size. The smaller the block degree is, the larger the surface area per unit volume is. The development direction and richness of the crack, and the roughness of the macroscopic failure surface, are also related to the failure energy.

### 5.2. Study on Failure Energy per Unit Area of Concrete Specimens

Since the concrete specimen is a composite material, the energy dissipation per unit area is calculated by studying the interface and mortar separately.

(12) $$U_{i}=U_{interface}+U_{mator}=\sum_{i=A}\omega_{1}+\sum_{i=B}\omega_{2}.$$

*i* is the number of calculation step; *U_interface_* is the interface energy; *U_mator_* is the mortar energy; *ω*_1_ is the strain energy of the interface element; *ω*_2_ is the strain energy of the mortar element; *A* is the number of interface elements; *B* is the number of mortar elements; *U_i_* is the total energy of each calculation step.

(13) $$\Delta U_{i}=U_{i}-U_{i-1}=\Delta U_{interface}+\Delta U_{mator.}$$

Δ*U_i_* is the energy loss between two loading steps when the load continues.

(14) $$\bar{\Delta u}=\frac{\sum_{i=n}\Delta U_{\mathrm{int}erface}(\sum_{i=n}\Delta U_{mator})}{\sum_{i=n}N(\sum_{i=n}M)}.$$

$\bar{\Delta U}$ is the unit destruction energy, *N* is the number of interface units killed per step, *M* is the number of mortar units killed per step, and *n* is the total number of loading steps.

(15) $$\bar{\Delta S}=\bar{\Delta U}/S.$$

Through the above calculations, the total damage energy and the energy per unit damage area can be obtained, as shown in Table 4. No matter what the loading mode and the height to diameter ratio of the specimen change, the loss per unit failure area is basically the same, at 2.0–6.0 mJ/cm^2^. Table 4 shows that, when the specimen is damaged by force, the failure surface is caused by overcoming the surface energy of the specimen, and the ability of the specimen itself to resist external load is called its strength.

According to the theoretical calculation, the relationship between the failure area and failure energy of specimens under different loading methods is determined:

*J_D_* = *S_D_ W*. (16)

*J_D_* is the destructive energy, *S_D_* is the destructive area, and *W* is the destructive energy per unit area.

In the numerical calculation, due to the influence of the specimen size effect, the influence of aggregate random position, the division of unit length in calculation, the difference of incremental steps, the selection of loading steps in displacement loading control, and the multiple splitting technology carried out in numerical calculation due to the thickness of interface elements, the failure energy of interface elements and mortar units of each model is different. The average unit failure energy of each model is also different, and the numerical calculation results are also different, but the failure energy per unit area is similar, and *W* is taken as a range value.

**Figure 9:**
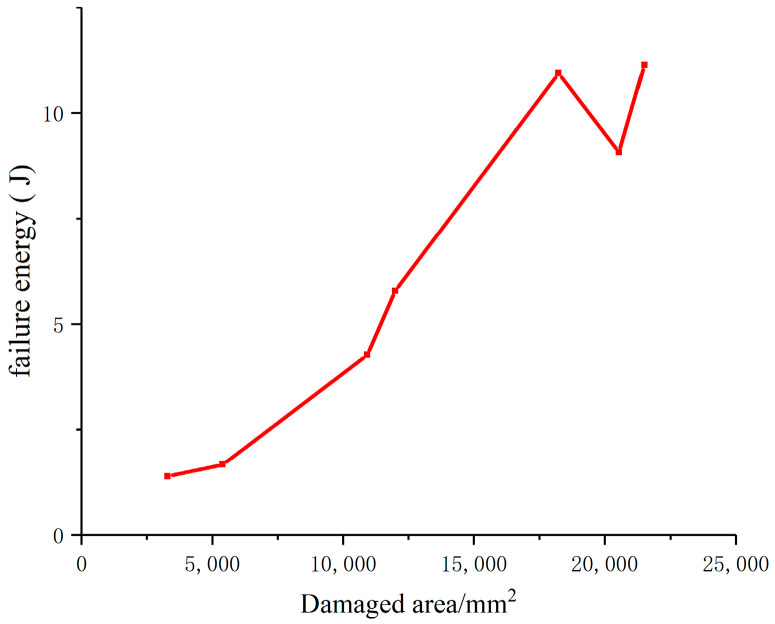
Curve of failure energy variation of specimen with failure area.

As can be seen from Figure 9, the failure energy and failure area corresponding to different loading methods in numerical calculation are proportional to each other, and the slope of the curve of failure energy and failure area is the failure energy per unit area; that is, the failure energy per unit area is the same, regardless of whether the loading method is pull, pressure (long-pressure, medium-pressure or-short pressure) or other loading methods. Formula (16) is further verified.

This theory is also consistent with Rittinger’s new surface energy theory, which holds that the physical and mechanical properties of rocks before and after crushing do not change, and only new surfaces are added after crushing. The surface energy of the new surface is proportional to the external energy input, and the energy lost during the crushing process is proportional to the new surface area.

The failure energy of concrete specimens varies linearly with the failure area, regardless of the loading method, and the failure energy per unit area is related to the physical properties of concrete materials. Different mix ratios, different aggregates and different cement grades lead to different failure energy per unit area.

However, this experiment only considers plain concrete, and does not study the damage area and energy of reinforced concrete or high-strength concrete, which need to be studied and analyzed in the future.

## 6. Conclusions

In order to study the strength changes of concrete specimens under different loading conditions, based on the principle of minimum energy dissipation and from the perspective of concrete failure energy per unit area, experiments and finite element numerical simulation were carried out on concrete specimens with different failure modes of tension, compression, bending and torsion, and the failure forms of concrete specimens under different loading conditions, as well as the failure area and failure energy of each specimen during loading, were analyzed. The failure energy per unit area under each failure mode is quantitatively calculated. The main conclusions are as follows:

(1) In the numerical simulation procedure, the fracture process of concrete specimens is considered as elastic–brittle failure. By further studying the relationship between the failure energy consumed by each specimen under different loading conditions and the failure area, the relationship equation between the two is established, and it is concluded that the failure energy per unit of the failure area, that is, the unit fracture energy *W*, is a constant during the fracture process of concrete materials. The failure energy per unit area is related to the physical properties of concrete materials. Different mix ratios, different aggregates and different cement grades lead to different failure energy *W* per unit area, which is consistent with Rittinger’s new surface energy theory.
(2) The failure process of concrete specimens is a process of overcoming material continuity and generating a new surface. When the concrete specimens break per unit area, a new surface of twice the area will be generated, and the surface energy inside the material overcome by the new surface is the energy consumed per unit area of damage. Therefore, the larger the failure area of the specimen, the more strain energy stored in the material is consumed, the more energy the specimen absorbs from the outside world, and the better the specimen is able to bear external work, due to its greater bearing capacity and greater strength.

## Figures and Tables

**Table 1:** Concrete specimen model under loading condition.

Model	Pull-1	Pre-(1)	Pre-(2)	Pre-(3)	Pre-(4)	Pre-(5)	Bending-1	Twist-1
(R × H) mm (L × B × H) mm	30 × 120	30 × 30	30 × 60	30 × 120	30 × 180	30 × 300	30 × 120	30 × 120

**Table 2:** Failure of compression specimens.

Aspect Ratio *λ*	≤1	1~10	≥10
Failure mode	Brittle failure	Brittle failure	Brittle failure
Failure pattern of specimen	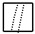	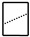	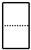
Failure mechanism	Shear cable-stayed failure	Shear failure predominates	Bending failure

**Table 3:** Failure area and failure energy of specimens under different loading conditions.

Different Loading Forms	Pull-1	Pre-1	Pre-3	Pre-5	Twist-1	Bending-1
Damaged area *S_D_* (m^2^)	0.002,826	0.025,43	0.016	0.002,826	0.002,115 9	0.002,826
Failure energy *U* (J)	0.002,826 R	0.025,43 R	0.016 R	0.002,826 R	0.002,115 9 R	0.002,826 R

**Table 4:** Strength and failure energy of different specimens.

Model	Strength (MPa)	Unit Average Failure Energy (J)	Failure Energy (J)	Number of Damaged Elements	Failure Energy per Unit Area (MJ/cm^2^)
Pull-1	7.56	4.29 × 10^−5^	0.5	Sj:34,069 Jm:11,033 45,212	2.02
Pre-1	29.78	2.78 × 10^−4^	11	Sj:14,892 Jm:26,356 41,248	5.24
Pre-2	22,287.7	1.78 × 10^−4^	10.8	Sj:12,041 Jm:62,8 98 74,939	5.38
Pre-3	18,409.1	1. 07 × 10^−4^	8.82	Sj:13,002, Jm:46,391 59,393	5.06
Pre-4	14,098.4	2.03 × 10^−4^	5.233 5	Sj:36,110 Jm:27,792 63,802	4.98
Pre-5	15,378.0	5.31 × 10^−5^	3.671 96	Sj:12,041 Jm:62,674 74,715	4.93
Bending-1	6.2	3.25 × 10^−5^	1.5	SJ:3,088 Jm:14,442 17,530	2.17

## Data Availability

Data are contained within the article.
